# A 3D texture dataset of 27 km road

**DOI:** 10.1016/j.dib.2021.106855

**Published:** 2021-02-09

**Authors:** Malal Kane

**Affiliations:** Université Gustave Eiffel, Campus de Nantes, Allée des Ponts et Chaussées, 44340 Bouguenais, France

**Keywords:** Road surface topography, LiDAR, Road-profile system

## Abstract

This dataset represents the 3D road surface texture of a route of 27 km long in a resolution of 0.1 m approximatively in both longitudinal and transversal directions. The first purpose of the dataset is to test numerical programs that need road surface topographies as input. The dataset is composed of 2658 text files and each representing a section of that route. The data was collected with the Harris2, a vehicle operated by TRL (Transport Research Laboratory from the UK), equipped with a LiDAR (Light Detection and Ranging) and a PPS (Road-Profile System). The files are presented in a regular grid achieved by merging the LIDAR and the PPS data into 3D coordinates.

## Specifications Table

**Table:** 

Subject	Civil and Structural Engineering
Specific subject area	Road monitoring
Type of data	Table (text files)
How data were acquired	The data were collected using the survey vehicle HARRIS2 operated by TRL. The vehicle is fitted with a Road-Profile System (PPS) high-resolution transverse profile laser, and a rotating LiDAR head to obtain point cloud.
Data format	The dataset is composed of 2658 text files. Each file representing a section of about 10 m long of that route with, and named with, the number of the section followed by the extension (Example: “0.txt, 1.txt, 2.txt … n.txt”). $\begin{array}{c} x_{1}y_{1}z_{x_{1},y_{1}}\\ x_{1}\vdots \vdots \\ x_{1}y_{n}z_{x_{1},y_{n}}\\ x_{2}y_{1}z_{x_{2},y_{1}}\\ x_{2}\vdots \vdots \\ x_{2}y_{n}z_{x_{2},y_{n}}\\ \vdots \vdots \vdots \\ x_{n}y_{1}z_{x_{n},y_{1}}\\ x_{n}\vdots \vdots \\ x_{n}y_{n}z_{x_{n},y_{n}} \end{array}$
Parameters for data collection	x and y represent the coordinates to localize a given point on the plane (x,y) and z the height of that point. x, y, z are in m. x- and y-directions are respectively the longitudinal and the transversal of the route with resolutions 0.0976 m and 0.0999 m approximatively.
Description of data collection	The road surface is discretized with a regular mesh. The data represent the heights of each of the nodes of this surface located by the coordinates (x,y).
Data source location	https://data.mendeley.com/datasets/b4cvrb7f8g/1
Data accessibility	No restriction, just download the zip file via the link https://data.mendeley.com/datasets/b4cvrb7f8g/1 and unzipped to get 2658 text files representing each section of about 10 m long and 4.2 m width. [1], “A 3D road surface topography dataset of an entire route”, Mendeley Data, V1, https://doi.org/10.17632/b4cvrb7f8g.1
Related research article	M. Kane, Road Safety: First step of an Algorithm to Identify the Potential Water Ponding on Routes, January 2021, Measurement, https://doi.org/10.1016/j.measurement.2021.108980

## Value of the Data

• This dataset is giving the topography of a road surface over an entire route with such a high resolution.
• This dataset is useful to test numerical programs (such as the potential of water ponding of a route or any or drive further investigations, which need road surface topographies as inputs).

## 1 Data Description

This dataset is composed of 2658 text files and each representing a section of a route of about 27 Km long. The files in a regular grid achieved by merging the LIDAR and the PPS data into 3D coordinates. The road surface is discretized with a regular mesh. The data represent the heights (z) of each of the nodes of this surface located by the coordinates (x,y). x and y represent the coordinates to localize a given point on the plane (x,y) and z the height of that point. x, y, z are in m. x- and y-directions are respectively the longitudinal and the transversal of the route with resolutions 0.0976 m and 0.0999 m approximatively. The data is represented in each of the files as follows:

$$\begin{array}{ccc} x_{1} & y_{1} & z_{x_{1},y_{1}}\\ x_{1} & \vdots & \vdots \\ x_{1} & y_{n} & z_{x_{1},y_{n}}\\ x_{2} & y_{1} & z_{x_{2},y_{1}}\\ x_{2} & \vdots & \vdots \\ x_{2} & y_{n} & z_{x_{2},y_{n}}\\ \vdots & \vdots & \vdots \\ x_{n} & y_{1} & z_{x_{n},y_{1}}\\ x_{n} & \vdots & \vdots \\ x_{n} & y_{n} & z_{x_{n},y_{n}} \end{array}$$

Each file of about 10 m long, and is named with the number of the section followed by the extension (Example: “0.txt, 1.txt, 2.txt … n.txt”).

## 2 Experimental Design, Materials and Methods

**Fig. 1:**
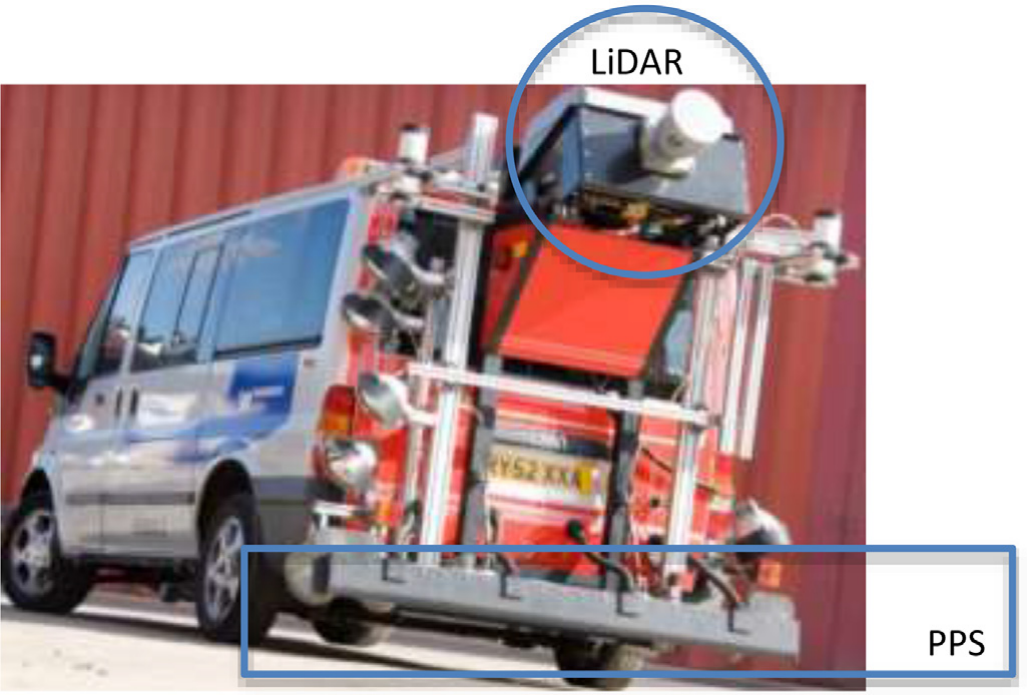
HARRIS2 vehicle with PPS laser and LIDAR attached.

The road data was collected using the survey vehicle HARRIS2 (Fig. 1) operated by TRL on a route of 27 km long located in the UK. The vehicle is fitted with a Road-Profile System (PPS) high-resolution transverse profile laser, and a rotating LiDAR to obtain point clouds. The raw data collected is not presented on a regular grid and must be resampled first to be used in the automated program. To do so, the raw LiDAR data must be translated into a reference frame that can be compared with those provided by the PPS [2].

## CRediT Author Statement

**Malal Kane**: Writing - Original draft preparation, data formatting, software development.

## Declaration of Competing Interest

Malal Kane declares that he has no known competing financial interests or personal relationships, which have or could be perceived to have influenced the work reported in this article.
